# Soft - sensing modeling based on ABC - MLSSVM inversion for marine low - temperature alkaline protease MP fermentation process

**DOI:** 10.1186/s12896-020-0603-x

**Published:** 2020-02-18

**Authors:** Bo Wang, Meifang Yu, Xianglin Zhu, Li Zhu

**Affiliations:** School of Electrical and Information Engineering, JiangSu University, Zhenjiang, 212013 Jiangsu China

**Keywords:** Marine alkaline protease MP, Material balance, Inverse system, Support vector machine, Soft-sensing

## Abstract

**Background:**

Aiming at the characteristics of nonlinear, multi-parameter, strong coupling and difficulty in direct on-line measurement of key biological parameters of marine low-temperature protease fermentation process, a soft-sensing modeling method based on artificial bee colony (ABC) and multiple least squares support vector machine (MLSSVM) inversion for marine protease fermentation process is proposed.

**Methods:**

Firstly, based on the material balance and the characteristics of the fermentation process, the dynamic “grey box” model of the fed-batch fermentation process of marine protease is established. The inverse model is constructed by analyzing the inverse system existence and introducing the characteristic information of the fermentation process. Then, the inverse model is identified off-line using MLSSVM. Meanwhile, in order to reduce the model error, the ABC algorithm is used to correct the inverse model. Finally, the corrected inverse model is connected in series to the marine alkaline protease MP fermentation process to form a composite pseudo-linear system, thus, real-time on-line prediction of key biological parameters in fermentation process can be realized.

**Results:**

Taking the alkaline protease MP fermentation process as an example, the simulation results demonstrate that the soft-sensing modeling method can solve the real-time prediction problem of key biological parameters in the fermentation process on-line, and has higher accuracy and generalization ability than the traditional soft-sensing method of support vector machine.

**Conclusions:**

The research provides a new method for soft-sensing modeling of key biological parameters in fermentation process, which can be extended to soft-sensing modeling of general nonlinear systems.

## Background

Marine alkaline protease MP is a fermentation enzyme that adapts well to the low-temperature environment [1]. In addition to a wide range of pH, the MP enjoys a high activity at room temperature, and becomes less active with the decrease of temperature [2, 3]. This emerging industrial enzyme brings new vitality and opportunities to such fields as medicine, food, enzyme industry, national defense and so on, and greatly broadens application regions of protease [4]. The marine low-temperature alkaline protease MP fermentation process is a dynamic process with high nonlinearity and strong coupling effect. Like general nonlinear systems, fermentation has time-varying, correlated and uncertain parameters [5]. Therefore, it is very difficult to directly measure the key biological parameters in the fermentation process [6]. Currently, these parameters can only be obtained through regular sampling, offline analysis and lab test. The current method has a poor real-time performance, and increases the bacteria exposure of the samples, hindering the advanced control of fermentation. This calls for a strategy that timely acquires the state of key biological parameters in the fermentation process. Without the state information, it is impossible to achieve dynamic and optimal control of the fermentation process, which boosts the biomass density and productivity of enzyme.

The inverse system method provides a good solution to the soft-sensing modelling of the fermentation process. This method boasts strict theoretical bases and clear physical meanings. Coupled with learning algorithms (e.g. neural network (NN) and support vector machine (SVM)), the inverse system method can complete soft-sensing of nonlinear systems, which are difficult to be modelled accurately [7, 8]. Suffice it to say that the inverse system method greatly facilitates the soft-sensing modelling of highly nonlinear systems in engineering practices. However, the inverse system method faces two problems in soft-sensing of the marine low-temperature alkaline protease MP fermentation process. On the one hand, the mathematical model of the controlled object and the system parameters of the model must be known before using the inverse system method. It is no easy task to obtain either information from the highly nonlinear and strongly coupled fermentation process. On the other hand, the inverse system of the original system must be established before using the inverse system method. In other words, the inverse system should be expressed mathematically in advance (that is, to derive a mathematical expression that can be used to describe the inverse system) [8]. To solve the problems, the literature [9] proposes a neural network inverse system method, which integrates intelligent control with the inverse system method. The inverse system is approximated by the neural network in this literature. The method was successfully applied to the soft-sensing of erythromycin fermentation, creating a “gray-box” model of the fermentation process. Nevertheless, the “gray-box” model is a simplified model based on the Monod equation, which ignores many important nonlinear factors in the actual process of erythromycin fermentation. Besides, the neural network, inspired by the asymptotic theory, is based on the unrealistic assumption that the number of samples is infinite, but the number of samples in the actual problem is often limited, especially the strong coupling, large lag complex nonlinear system as the marine alkaline protease MP fermentation process, it is extremely difficult to obtain accurate sample data. Therefore, in the case of small samples, the research of inverse soft-sensing methods suitable for the marine alkaline protease MP fermentation process and easy to implement in engineering has become the key problem to be solved urgently in the marine low-temperature alkaline protease MP fermentation process.

Considering the limited number of samples in actual fermentation, this paper attempts to design an easy-to-use inverse system method for soft-sensing modeling of the marine alkaline protease MP fermentation process. Firstly, a “gray-box” dynamic model was established for the the marine low-temperature alkaline protease MP fermentation process, according to material balance and features of that process. Secondly, the existing inverse system was analyzed, and the design of extended inverse model was introduced. Thirdly, the offline identification of MLSSVM and online optimization of ABC were combined to develop the extended inverse model based on ABC-MLSVM, and the extended inverse model was connected in series after the primary fermentation process, serving as the soft-sensing model that predicts key biological parameters online in real time. Fourthly, the effectiveness of the soft-sensing modelling method was verified through a simulation of the MP fermentation in lab; the simulation results show that the method can effectively predict the key biological parameters of the marine low-temperature alkaline protease MP fermentation process online, and outperform the traditional least square support vector machine (LS-SVM) soft-sensing modeling method in prediction accuracy.

## Methods

### Dynamic model of fermentation process

In this paper, Taking the fermentation process as the object, this paper assumes that both cell concentration and the protease concentration are zero. The fermentation states (concentration of each substance) were taken as dependent variables of differential equation, while time *t* was taken as an independent variable or separate variable. Then, the “grey box” dynamic model could be described by the material balance equations of various substances (mycelia, restrictive substrate, protease, oxygen, H+, etc.) [10], as shown in Eq. (1):

1 $$ \mathrm{Variable}\ \mathrm{quantity}\left(\mathrm{Target}\ \mathrm{substance}\ \mathrm{per}\ \mathrm{unit}\ \mathrm{of}\ \mathrm{time}\right)=\mathrm{influx}\left(\mathrm{Target}\ \mathrm{substance}\ \mathrm{per}\ \mathrm{unit}\ \mathrm{of}\ \mathrm{time}\right)-\mathrm{outflow}+\mathrm{formation}\ \mathrm{amount} $$

The construction process of the dynamic model of the ash box is as follows:

#### Volume change equilibrium equation

During the fermentation process, culture medium should be added at a rational rate to supplement the nutrients and increase the protease yield. The culture medium mainly consists of carbon source, nitrogen source, inorganic salt, growth factor and enzyme-producing promoter. Through preliminary experiments, this paper selects maize flour hydrolysate as carbon source, soybean meal hydrolysate as nitrogen source, ammonia sulfate ((NH_4_)_2_SO4) as inorganic salt, malt extract as growth factor and Polysorbate 80 (Tween-80) as surfactant. The volume (*V*) of the fermentation broth changes with the addition of nutrients and enzyme-producing promoter. The equilibrium equation is as follows:

2 $$ \frac{\mathrm{d}V}{\mathrm{d}t}={f}_{mh}+{f}_s+{f}_a+{f}_m+{f}_{tw} $$

where: *V* is fermentation broth volume, *f*_*mh*_, *f*_*s*_, *f*_*a*_, *f*_*m*_ and *f*_*tw*_ are respectively the flow rate of maize flour hydrolysate, soyabean cake meal hy-drolysate, (NH_4_)_2_SO_4_), malt extract and enzyme-producing promoter (Tween-80).

#### Cell growth kinetics equation

The previous studies have found that, the growth of the enzyme producing strain has a maximum concentration, i.e. a saturation point, which could be reached if the initial sugar concentration is on suitable levels. The time to reach the saturation point varies with the initial sugar concentration. The higher the initial sugar concentration, the slower the cells grow, that is, the substrate concentration inhibits the cell growth. Considering the deviation of Monod equation-based description, the logistic equation was employed to depict the growth law of the cells, in the light of the volume change in fed-batch fermentation, and the volume change during fed-batch fermentation is taken into account. The growth kinetics model of cell is as follows:

3 $$ \frac{dX}{dt}=\mu X-\frac{X}{V}\frac{dV}{dt}\kern0.2em $$

where: *μ* is the specific growth rate of somatic cells, *X* is cell concentration.

#### Substrate consumption equation

The substrate consumption of marine low-temperature alkaline protease MP was modelled based on the material balance. The effect of additive carbon source (maize flour hydrolysate) was considered in the model, because the carbon source, as the only restrictive substrate, is consumed rapidly in large quantities. The model is expressed as follows:

4 $$ \frac{dS}{dt}\kern0.5em =-\nu X+\frac{S_{mh}}{V}-\frac{S}{V}\frac{dV}{dt}\kern0.1em $$

where: *S* is the substrate concentration, *ν* is the specific consumption rate of substrate (*h*^−1^), *S*_*mh*_ is the maize flour hydrolysate flow rate.

#### Protease synthesis kinetics

The model of fermentation enzyme production is partial growth coupled type (It belongs to extracellular enzyme, and its synthesis regulation is affected by many mechanisms), high concentration substrate can obviously inhibit the secretion of protease while maintaining low carbon source concentration is beneficial to the secretion of protease MP. At the same time, the hydrolysis of alkaline protease MP also has a certain effect on protease MP itself. On this basis, Tween-80, growth factor and the hydrolysis rate which have influence on the fermentation process are introduced into the protease synthesis kinetics, the model is expressed as follows:

5 $$ \frac{\mathrm{d}E}{\mathrm{d}t}=\rho X- KP+\frac{K_m}{V}{f}_m+\kern0.3em \frac{K_{tw}}{V}{f}_{tw}-\frac{E}{V}\frac{\mathrm{d}V}{\mathrm{d}t} $$

where: *E* is protease content (%), *ρ* is the specific growth rate of protease, *K is* hydrolysis constants of protease, and *K*_*m*_, *K*_*tw*_ are inhibition constants.

#### Dissolved oxygen concentration (DO) variation model

The MP fermentation is aerobic, i.e. oxygen is involved in cell growth and protease synthesis. DO must be controlled in a suitable range. In fact, the oxygen demand constantly changes through the cell growth, because cell concentration and cell respiration intensity change from stage to stage. Based on the varying oxygen demands, the DO in the fermentation broth must be regulated in real time. According to the aerobic features of MP fermentation and the effect of bioreactor size on DO level in culture medium, the oxygen volumetric mass transfer coefficient of the bioreactor was introduced to the DO concentration equilibrium equation. The equilibrium equation is as follows:

6 $$ \frac{\mathrm{d}{C}_L}{\mathrm{d}t}=-\eta X+{K}_{La}\left({C}_L^{\ast }-{C}_L\right)-\frac{C_L}{V}\frac{\mathrm{d}V}{\mathrm{d}t} $$

where: *C*_*L*_ is the DO concentration (mol/*L*), *K*_*La*_ is oxygen volume mass transfer coefficient in bioreactor (*s*^−1^), ${C}_L^{\ast }$ is saturation concentration of oxygen dissolved in fermentation liquid phase (mol/*L*), and *η* is the specific consumption rate of oxygen.

#### PH dynamic change model

During the fermentation, the enzyme producing strain favors an alkaline environment, with the optimal pH range of 9.0~10.0. Any change of pH in fermentation broth will exert a huge impact on the fermentation of the MP. Excessively high or low pH values will slow down the strain growth and the formation of protease, weakening the enzyme activity. Therefore, the pH of fermentation broth was regulated within the optimal range by the flow of nutrients through the fermentation process, so that it can be maintained in the optimum range. The pH equilibrium equation of fermentation broth is expressed as:

7 $$ \frac{\mathrm{d}{\left[\mathrm{H}\right]}^{+}}{\mathrm{d}t}=\gamma X-\frac{{\left[\mathrm{H}\right]}^{+}}{V}\frac{\mathrm{d}V}{\mathrm{d}t}+\frac{S_s{f}_s-{S}_{mh}{f}_{mh}-{S}_m{f}_m-{S}_{tw}{f}_{tw}}{V} $$

where:[H]^+^ is the hydrogen ion concentration in fermentation broth (used to characterize the pH of fermentation broth), *f*_*mh*_, *f*_*s*_, *f*_*m*_ and *f*_*tw*_ are respectively flow rate of maize flour hydrolysate, soyabean cake meal hy-drolysate, malt extract and Tween-80. *S*_*mh*_, *S*_*s*_, *S*_*m*_, *S*_*tw*_ are respectively the liquid concentrations of maize flour hydrolysate, soyabean cake meal hy-drolysate, malt extract and Tween-80, *γ* is the specific consumption of [H+].

Through the above analysis, the “gray-box” dynamic model of the marine low-temperature alkaline protease MP fermentation process can be expressed as:

8 $$ \Big\{{\displaystyle \begin{array}{l}{\dot{x}}_1=\mu {x}_1-\frac{x_1}{x_6}\sum \limits_{i=1}^5{u}_i\\ {}{\dot{x}}_2\kern0.5em =-\nu {x}_1+\frac{s_1{u}_1}{x_6}-\frac{x_2}{x_6}\sum \limits_{i=1}^5{u}_i\\ {}{\dot{x}}_3\kern0.4em =\rho {x}_1-{s}_2{x}_3+\frac{s_3{u}_4}{x_6}+\frac{s_4{u}_5}{x_6}-\frac{x_3}{x_6}\sum \limits_{i=1}^5{u}_i\\ {}{\dot{x}}_4=-\eta {x}_1-{s}_5{x}_4+{s}_6-\frac{x_4}{x_6}\sum \limits_{i=1}^5{u}_i\\ {}{\dot{x}}_5=\gamma {x}_1+\frac{s_7{u}_2-{s}_1{u}_1-{s}_8{u}_4-{s}_9{u}_5}{x_6}-\frac{x_5}{x_6}\sum \limits_{i=1}^5{u}_i\\ {}{\dot{x}}_6=\sum \limits_{i=1}^5{u}_i={u}_1+{u}_2+{u}_3+{u}_4+{u}_5\end{array}} $$

where: **x** = [*x*_1_, *x*_2_, *x*_3_, *x*_4_, *x*_5_, *x*_6_]^T^ = [*X*, *S*, *E*, *C*_L_, [H]^+^, *V*]^T^ represent the status vector, **u** = [*u*_1_, *u*_2_, *u*_3_, *u*_4_, *u*_5_]^T^ = [*f*_*mh*_, *f*_*s*_, *f*_*a*_, *f*_*m*_, *f*_*tw*_]^T^ is the input vector, *μ*(**x**), *ν*(**x**), *ρ*(**x**), *η*(**x**), *γ*(**x**) are the analytical functions of the respective status variables **x**, *S*_*i*_ (*i* = 1, 2, ⋯9) are all constants other than zero and represent respectively the liquid feeding concentration of maize flour hydrolysate, hydrolysis constants of protease, inhibition constant, gas saturated oxygen concentration, ${C}_L^{\ast}\ast {K}_{La}$, the liquid feeding concentration of soyabean cake meal hy-drolysate, (NH_4_)_2_SO_4_ and Tween-80.

### Reversibility analysis

**Fig. 1:**
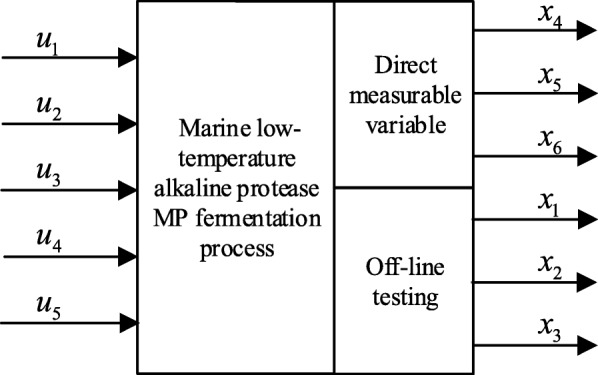
*u*_1_~*u*_5_ are input parameters, *x*_1_~*x*_6_ are the six process parameters of fermentation process. The process parameters *x*_4_, *x*_5_, *x*_6_ are directly measurable parameters and *x*_1_, *x*_2_, *x*_3_ are key parameters that are difficult to measure directly on-line (That is, the variable that needs to be predicted)

The marine low-temperature alkaline protease MP fermentation process is shown in Fig. 1. *u*_1_~*u*_5_ are input parameters, *x*_1_~*x*_6_ are the six process parameters of fermentation process. The process parameters *x*_4_, *x*_5_, *x*_6_ are directly measurable parameters and *x*_1_, *x*_2_, *x*_3_ are key parameters that are difficult to measure directly on-line (That is, the variable that needs to be predicted).

**Fig. 2:**
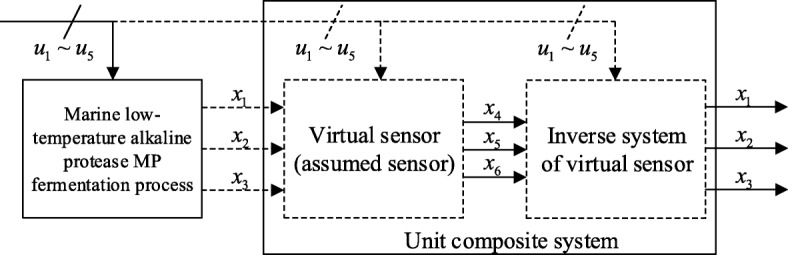
This “virtual subsystem” is regarded as a “virtual sensor” in the marine low-temperature alkaline protease MP fermentation process. It can be used as a dynamic compensator in series with the “virtual subsystem”, a unit compound system can be formed. Its the input and output present an identity mapping relationship, that is, the output of the composite system can completely reproduce the input of the original “virtual sensor”, and then the soft-sensing of *x*_1_, *x*_2_, *x*_3_ can be realized

In order to predict the non-direct measurable key biological parameters *x*_1_, *x*_2_, *x*_3_, a virtual subsystem was assumed to exist in the marine low-temperature alkaline protease MP fermentation process, including three non-directly measureable inputs *x*_1_, *x*_2_, *x*_3_, three directly measurable outputs *x*_4_, *x*_5_, *x*_6_ and five variables *u*_1_~*u*_5_. This virtual subsystem is regarded as a “virtual sensor” [11] of the marine low-temperature alkaline protease MP fermentation process. The soft-sensing of *x*_1_, *x*_2_, *x*_3_ can be realized through the following steps: solve the inverse model of the virtual subsystem; take the model as a dynamic compensator in series with the virtual subsystem, forming a composite system [12]; reproduce the inputs of the “virtual sensor” based on the outputs of the composite system, as shown in Fig. 2.

In order to achieve the soft-sensing of key biological parameters *x*_1_, *x*_2_, *x*_3_, the reversibility analysis of the “virtual sensor” must be carried out, and the inverse system model should be solved.

**Lemma***Necessary and sufficient conditions for system Σ reversibility in some field of point* (*x*_0_, *u*_0_): *The system meets*$\mathit{\operatorname{rank}}\left(\partial {z}_m^{\mathrm{T}}/\partial {\hat{\mathbf{x}}}^{\mathrm{T}}\right)={r}_m=l$ , *l is the dimension number of non-direct measurable variable*.

The reversibility of “virtual sensor” is analyzed by ***Interactor*** algorithm: The direct measurable variable **z** = [*z*_1_, *z*_2_, *z*_3_] = [*x*_4_, *x*_5_, *x*_6_] are derived by using the modeling algorithm, and all-order derivatives ${\dot{z}}_i,{\ddot{z}}_i,\cdots, {z}_i^{\left({k}_i\right)}$ (*i* = 1, 2, 3) can be obtained, and then the independent derivative information of the function is selected to form the vector **Z**_*m*_,as shown in Eq. 9:

9 $$ \Big\{{\displaystyle \begin{array}{l}{\dot{x}}_4=-\eta {x}_1-{s}_5{x}_4+{s}_6-\frac{x_4}{x_6}\sum \limits_{i=1}^5{u}_i\\ {}{\ddot{x}}_4={g}_1\left(\mathbf{x},\mathbf{u}\right)+{g}_2\left({x}_4,{x}_5,{x}_6,\mathbf{u},\dot{\mathbf{u}}\right)\\ {}{\dot{x}}_5=\gamma {x}_1+\frac{s_7{u}_2-{s}_1{u}_1-{s}_8{u}_4-{s}_9{u}_5}{x_6}-\frac{x_5}{x_6}\sum \limits_{i=1}^5{u}_i\end{array}} $$

where:

10 $$ \Big\{{\displaystyle \begin{array}{l}{g}_1\left(\mathbf{x},\mathbf{u}\right)=\left(\frac{\partial \eta }{\partial {x}_1}{x}_1+\frac{\partial \eta }{\partial {x}_2}{x}_2+\frac{\partial \eta }{\partial {x}_3}{x}_3+\frac{\partial \eta }{\partial {x}_4}{x}_4+\frac{\partial \eta }{\partial {x}_5}{x}_5\right)\frac{x_1}{x_6}\sum \limits_{i=1}^5{u}_i\\ {}+{s}_5\eta {x}_1-\left(\frac{\partial \eta }{\partial {x}_1}\mu -\frac{\partial \eta }{\partial {x}_2}\nu +\frac{\partial \eta }{\partial {x}_3}\rho -\frac{\partial \eta }{\partial {x}_4}\eta +\frac{\partial \eta }{\partial {x}_5}\gamma \right){x}_1^2\\ {}+\frac{\partial \eta }{\partial {x}_3}\kern0.3em {s}_2\kern0.1em {x}_1\kern0.1em {x}_3\kern0.3em -\kern0.32em \frac{\partial \eta }{\partial {x}_3}\frac{x_1}{x_6}\kern0.1em \left(\kern0.1em {s}_3{u}_4+{s}_4{u}_5\right)-\eta \kern0.1em \mu \kern0.2em {x}_1-\kern0.3em \frac{\partial \eta }{\partial {x}_4}\kern0.3em {s}_6\kern0.3em {x}_1\kern0.5em \\ {}+\kern0.3em \frac{\partial \eta }{\partial {x}_5}\frac{x_1}{x_6}\kern0.1em {s}_8{u}_4\kern0.3em -\frac{\partial \eta }{\partial {x}_5}\kern0.3em \frac{x_1}{x_6}\kern0.1em {s}_7{u}_2-{s}_1\kern0.2em \left(\kern0.3em \frac{\partial \eta }{\partial {x}_2}\kern0.3em -\kern0.4em \frac{\partial \eta }{\partial {x}_5}\kern0.3em \right)\kern0.4em \frac{x_1}{x_6}\kern0.4em {u}_1\\ {}+\frac{\partial \eta }{\partial {x}_4}\kern0.3em {s}_5\kern0.2em {x}_1\kern0.2em {x}_4+\kern0.4em \frac{2\eta {x}_1}{x_6}\kern0.3em \sum \limits_{i=1}^5\kern0.3em {u}_i+\kern0.4em \frac{\partial \eta }{\partial {x}_5}\kern0.4em \frac{x_1}{x_6}\kern0.6em {s}_9\kern0.1em {u}_5\kern1.68em \\ {}{g}_2\left({x}_4,{x}_5,{x}_6,\mathbf{u},\dot{\mathbf{u}}\right)=2\frac{s_5{x}_4}{x_6}\sum \limits_{i=1}^5{u}_i-\frac{s_6}{x_6}\sum \limits_{i=1}^5{u}_i+{s}_5^2{x}_4-{s}_5{s}_6\\ {}+\kern0.3em 2\frac{x_4}{x_6^2}\kern0.5em {\left(\kern0.3em \sum \limits_{i=1}^5\kern0.2em {u}_i\kern0.2em \right)}^2-\kern0.4em \frac{x_4}{x_6}\kern0.5em \sum \limits_{i=1}^5{\dot{u}}_i\end{array}}\kern0.1em $$

According to Eq. (9), $\partial {\ddot{z}}_2/\partial {x}_i=\partial {g}_1(x)/\partial {x}_i,i=1,2,3,$.

*m* = 3is obtained, and the Jacobian Matrix $J=\partial {\left({\ddot{z}}_1,{\dot{z}}_1,{\dot{z}}_2\right)}^T/\partial \left({x}_1,{x}_2,{x}_3\right)$ is further solved:

11 $$ J=\left[\begin{array}{ccc}\frac{\partial {\ddot{z}}_1}{\partial {x}_1}& \frac{\partial {\ddot{z}}_1}{\partial {x}_2}& \frac{\partial {\ddot{z}}_1}{\partial {x}_3}\\ {}\frac{\partial {\dot{z}}_1}{\partial {x}_1}& \frac{\partial {\dot{z}}_1}{\partial {x}_2}& \frac{\partial {\dot{z}}_1}{\partial {x}_3}\\ {}\frac{\partial {\dot{z}}_2}{\partial {x}_1}& \frac{\partial {\dot{z}}_2}{\partial {x}_2}& \frac{\partial {\dot{z}}_2}{\partial {x}_3}\end{array}\right]=\left[\begin{array}{ccc}\frac{\partial {g}_1\left(\mathbf{x},\mathbf{u}\right)}{\partial {x}_1}& \frac{\partial {g}_1\left(\mathbf{x},\mathbf{u}\right)}{\partial {x}_2}& \frac{\partial {g}_1\left(\mathbf{x},\mathbf{u}\right)}{\partial {x}_3}\\ {}-\frac{\partial \eta }{\partial {x}_1}{x}_1-\eta & -\frac{\partial \eta }{\partial {x}_2}{x}_1& -\frac{\partial \eta }{\partial {x}_3}{x}_1\\ {}\frac{\partial \gamma }{\partial {x}_1}{x}_1+\gamma & \frac{\partial \gamma }{\partial {x}_2}{x}_1& \frac{\partial \gamma }{\partial {x}_3}{x}_1\end{array}\right] $$

After the transformation of the elementary row of Eq. (11) and obtain the following:

12 $$ \tilde{\mathbf{J}}=\left[\begin{array}{lll}\begin{array}{l}{g}_7\left(\mathbf{x},\mathbf{u}\right)\\ {}\end{array}& 0& 0\\ {}\begin{array}{l}{g}_3\left(\mathbf{x},\mathbf{u}\right)\\ {}\end{array}& {g}_4\left(\mathbf{x},\mathbf{u}\right)& 0\\ {}\frac{\partial \gamma }{\partial {x}_1}{x}_1+\gamma & \frac{\partial \gamma }{\partial {x}_2}{x}_1& \frac{\partial \gamma }{\partial {x}_3}{x}_1\end{array}\right] $$

where: $$ {g}_3\left(\mathbf{x},\mathbf{u}\right)=\left[\left(\frac{\partial \gamma }{\partial {x}_1}{x}_1+\gamma \right)\frac{\partial \eta }{\partial {x}_3}\right]/\frac{\partial \gamma }{\partial {x}_3}-\frac{\partial \eta }{\partial {x}_1}{x}_1-\eta $$;$$ {g}_4\left(\mathbf{x},\mathbf{u}\right)=\left({x}_1\frac{\partial \gamma }{\partial {x}_2}\frac{\partial \eta }{\partial {x}_3}\right)/\frac{\partial \gamma }{\partial {x}_3}-\frac{\partial \eta }{\partial {x}_2}{x}_2; $$$$ {g}_5\left(\mathbf{x},\mathbf{u}\right)=\frac{\partial {g}_1\left(\mathbf{x},\mathbf{u}\right)}{\partial {x}_1}- $$$$ \left[\left(\frac{\partial v}{\partial {x}_1}{x}_1+\gamma \right)\frac{\partial {g}_1\left(\mathbf{x},\mathbf{u}\right)}{\partial {x}_3}\right]/\frac{\partial v}{\partial {x}_3} $$; $$ {g}_6\left(\mathbf{x},\mathbf{u}\right)=\frac{\partial {g}_1\left(\mathbf{x},\mathbf{u}\right)}{\partial {x}_2}- $$$$ \left(\frac{\partial \gamma }{\partial {x}_2}\frac{\partial {g}_1\left(\mathbf{x},\mathbf{u}\right)}{\partial {x}_3}\right)/\frac{\partial \gamma }{\partial {x}_3} $$;

$$ {g}_7\left(\mathbf{x},\mathbf{u}\right)={g}_5\left(\mathbf{x},\mathbf{u}\right)-\frac{g_6\left(\mathbf{x},\mathbf{u}\right)}{g_4\left(\mathbf{x},\mathbf{u}\right)}{g}_3\left(\mathbf{x},\mathbf{u}\right). $$

If $\det \left(\overset{\sim }{J}\right)={g}_7\left(\boldsymbol{x},\boldsymbol{u}\right)\bullet {g}_4\left(\boldsymbol{x},\boldsymbol{u}\right)\bullet \frac{\partial \gamma }{\partial {x}_3}{x}_1\not\equiv 0$ in the entire real vector space, it can be known that $\mathbf{J}=\partial {\mathbf{Z}}_m^{\mathrm{T}}/\partial {\hat{\mathbf{x}}}^{\mathrm{T}}=\partial \left({\ddot{z}}_1,{\dot{z}}_1,{z}_2\right)/\partial \left({x}_1,{x}_2,{x}_3\right)=3$ from **Lemma**, and it meet the system reversibility condition, that is, the system is globally reversible. However, for $\det\ \left(\overset{\sim }{J}\right)\not\equiv 0$, it is quite difficult to guarantee that it satisfies the non-zero conditions everywhere in the entire real vector space ***R***.

Considering the above situation and the current operation state of the marine low temperature alkaline protease MP fermentation process, it is assumed that $\det\ \left(\overset{\sim }{J}\right)\not\equiv 0$, a small work area of the fermentation process within the real vector space *R*, satisfies the reversibility condition of the “virtual sensor”. Then, an inverse soft-sensing model is constructed based on ABC-MLSVM. The assumption is verified against actual analysis results.

Suppose the system satisfies the reversibility condition for the work area. Then, the inverse system of the virtual sensor of he marine low-temperature alkaline protease MP fermentation process is established based on the inverse function theorem, using Eqs. (8)~(9):

13 $$ \hat{\mathbf{x}}=\left(\begin{array}{l}{x}_1\\ {}{x}_2\\ {}{x}_3\end{array}\right)=\left(\begin{array}{l}{\varphi}_1\left({x}_4,{x}_5,{x}_6,{\dot{x}}_4,{\ddot{x}}_4,{\dot{x}}_5,\mathbf{u},\dot{\mathbf{u}}\right)\\ {}{\varphi}_2\left({x}_4,{x}_5,{x}_6,{\dot{x}}_4,{\ddot{x}}_4,{\dot{x}}_5,\mathbf{u},\dot{\mathbf{u}}\right)\\ {}{\varphi}_3\left({x}_4,{x}_5,{x}_6,{\dot{x}}_4,{\ddot{x}}_4,{\dot{x}}_5,\mathbf{u},\dot{\mathbf{u}}\right)\end{array}\right) $$

The “gray-box” model is obtained through lab research based on the material balance of fed-batch fermentation. The model ignores the influence of many factors, and merely approximates the actual kinetics [13]. There are several constraints of this model: (1) The temperature, fermenter pressure and agitation speed are constant in the fermentation process; (2) The broth and substrate concentrations are not affected by fermentation heat. The inverse system model Eq. (13) of the “virtual sensor” is established based on the gray box model under the above constraints. Obviously, the established model fails to reflect the influence of several key factors in the fermentation process, including but not limited to fermentation temperature, in-tank pressure, agitation speed, and air flowrate. As a result, the soft-sensing prediction based on Eq. (13) will have a huge error, undermining the subsequent optimization control.

To overcome the defect, fermentation temperature (*W*_*t*_), tank inside pressure(*P*_*t*_), agitation speed (*S*_*a*_), air flow rate (*F*_*a*_) four process parameters are included to the soft-sensing model based on Eq. (13). The structure of the extended inverse model for soft-sensing can be described as:

14 $$ \hat{\mathbf{x}}=\left(\begin{array}{l}{x}_1\\ {}{x}_2\\ {}{x}_3\end{array}\right)=\left(\begin{array}{l}{\varphi}_4\left({x}_4,{x}_5,{x}_6,{\dot{x}}_4,{\ddot{x}}_4,{\dot{x}}_5,\mathbf{u},\dot{\mathbf{u}},{W}_t,{P}_t,{S}_a,{F}_a\right)\\ {}{\varphi}_5\left({x}_4,{x}_5,{x}_6,{\dot{x}}_4,{\ddot{x}}_4,{\dot{x}}_5,\mathbf{u},\dot{\mathbf{u}},{W}_t,{P}_t,{S}_a,{F}_a\right)\\ {}{\varphi}_6\left({x}_4,{x}_5,{x}_6,{\dot{x}}_4,{\ddot{x}}_4,{\dot{x}}_5,\mathbf{u},\dot{\mathbf{u}},{W}_t,{P}_t,{S}_a,{F}_a\right)\end{array}\right) $$

The addition of the key parameters provides the extended inverse model for soft-sensing with more characteristic information of the fermentation process, which greatly promotes the adaptability and anti-jamming ability of the model.

Although the inverse soft-sensing model of the marine low-temperature alkaline protease MP fermentation process is constructed in this paper, However, Eq. (14) shows that the extended inverse model for soft-sensing is difficult to solve, despite the possible existence of a solution. The LSSVM offers a solution to this problem, thanks to its strong approximation ability to nonlinear functions.

### Improved MLSSVM

Traditional LSSVM is grounded on multi-input single-output (MISO) systems. It cannot be directly applied to identify multi-input multi-output (MIMO) systems [14], such as the extended inverse model of the marine alkaline protease MP fermentation process. Thus, the MLSSVM was proposed to build the extended inverse model of the marine alkaline protease MP fermentation process (MIMO).

LSSVM is proposed by Suykens in which author has changed the inequality constraints in SVM [15, 16] with equality and converted the convex quadratic programming problem to a convex linear system problem, which is often used for model decomposition problems and function prediction. Its modeling principle is as follows:

Given *l* training samples {(**x**_*i*_, *y*_*i*_)|   *i* = 1, 2, ..., *l*}, **x**_*i*_ ∈ *R*^*n*^ is an input and *y*_*i*_ ∈ *R* is output. The optimization problem for regression LSSVM is as follows:

15 $$ {\displaystyle \begin{array}{l}\min J\left(\mathbf{w},b,\xi \right)=\frac{1}{2}{\left\Vert \mathbf{w}\right\Vert}^2+\frac{1}{2}\gamma \sum \limits_{i=1}^l{\xi}_i^2\\ {}\mathrm{s}.\mathrm{t}.{y}_i={\mathbf{w}}^{\mathrm{T}}\varphi \left({\mathbf{x}}_i\right)+b+{\xi}_i\kern0.24em i=1,2,\cdots, l\end{array}} $$

where: **w** is the weight vector, *b* is the deviation, *φ*(**x**_*i*_) is a mapping to a high dimensional space, *ξ*_*i*_ is relaxation factor (error), *γ* is regularization parameter.

In order to transform the single-output optimization problem into a multi-output optimization problem. In this paper, the quadratic loss function of error(${\boldsymbol{\upxi}}_i{\boldsymbol{\upxi}}_i^{\mathrm{T}}$, **ξ** ∈ **R**^1 × *n*^) is introduced to replace the relaxation factor(${\xi}_i^2$) in optimization problem(15):

16 $$ {\displaystyle \begin{array}{l}\min J\left(\mathbf{w},b,\boldsymbol{\upxi} \right)=\frac{1}{2}\sum \limits_{i=1}^n{{\mathbf{w}}_i}^{\mathrm{T}}{\mathbf{w}}_i+\frac{1}{2}{\gamma}_i\sum \limits_{i=1}^n{\boldsymbol{\upxi}}_i{\boldsymbol{\upxi}}_i^{\mathrm{T}}\\ {}s.t.{\mathbf{y}}_i={\mathbf{w}}_i^{\mathrm{T}}{\varphi}_i\left(\mathbf{x}\right)+{\boldsymbol{\upgamma}}^{\mathrm{T}}{b}_i+{\boldsymbol{\upxi}}_i\kern0.24em i=1,2,\cdots, l\end{array}} $$

where: **ξ** ∈ **R**^1 × *n*^, *n* is the number of output variables, *φ*_*i*_(**x**) = [*φ*_*i*_(**x**_1_), ⋯, *φ*_*i*_(**x**_*l*_)].

Lagrange function is used to solve the above optimization problems:

17 $$ L=\frac{1}{2}\sum \limits_{i=1}^l{{\mathbf{w}}_i}^{\mathrm{T}}{\mathbf{w}}_i+\frac{1}{2}\sum \limits_{i=1}^l{\boldsymbol{\upxi}}_i{\boldsymbol{\upxi}}_i^{\mathrm{T}}-\sum \limits_{i=1}^l{\mathbf{a}}_i^{\mathrm{T}}\left({\mathbf{w}}_I^{\mathrm{T}}{\varphi}_i\left(\mathbf{x}\right)+{\boldsymbol{\upgamma}}^{\mathrm{T}}{b}_i+{\boldsymbol{\upxi}}_i-{\mathbf{y}}_i\right) $$

where: **a**_*i*_ ∈ **R**^*m* × *l*^ is a Lagrange multiplier, *m* is input vectors number.

According to the KKT condition, the transformation to linear equation is as follow:

18 $$ \Big\{{\displaystyle \begin{array}{l}\frac{\partial L}{\partial {\mathbf{w}}_i}=0\to {\mathbf{w}}_i=\varphi \left({\mathbf{x}}_i\right){\mathbf{a}}_i^{\mathrm{T}}\\ {}\frac{\partial L}{\partial {b}_i}=0\to {\boldsymbol{\upgamma}}^{\mathrm{T}}{\mathbf{a}}_i^{\mathrm{T}}=0\\ {}\frac{\partial L}{\partial {\boldsymbol{\upxi}}_i}=0\to {\mathbf{a}}_i={\boldsymbol{\upxi}}_i,i=1,2,\cdots, l\\ {}\frac{\partial L}{\partial {\mathbf{a}}_i}=0\to {\mathbf{w}}_i^{\mathrm{T}}{\varphi}_i\left(\mathbf{x}\right)+{\boldsymbol{\upgamma}}^{\mathrm{T}}{b}_i+{\boldsymbol{\upxi}}_i-{\mathbf{y}}_i=0,i=1,2,\cdots, l\end{array}} $$

From the above equations, ${\mathbf{w}}_i={\mathbf{a}}_i{\varphi}_i^{\mathrm{T}}\left(\mathbf{x}\right)$ and **ξ**_*i*_ = **a**_*i*_ can be easily obtained, and then they can be substituted into the last term of Eq. (18):

19 $$ {\mathbf{a}}_i{\varphi}_i^{\mathrm{T}}\left(\mathbf{x}\right){\varphi}_i\left(\mathbf{x}\right)+{\boldsymbol{\upgamma}}^{\mathrm{T}}{b}_i+{\mathbf{a}}_i-{\mathbf{y}}_i=0 $$

So that for the above optimization problem(18), the estimation function is written as:

20 $$ \left[{b}_i\kern0.5em {\mathbf{a}}_i\right]\left[\begin{array}{cc}0& {\boldsymbol{\upgamma}}^{\mathrm{T}}\\ {}\boldsymbol{\upgamma} & {K}_i\left({\mathbf{x}}_i,\mathbf{x}\right)+I\end{array}\right]=\left[0\kern0.5em {\mathbf{y}}_i\right] $$

where: *K*(**x**_*i*_, **x**) is the kernel function satisfying Mercer condition. In this paper, the kernel function is Gaussian radial basis function.

Considering that the matrix $$ \left[\begin{array}{cc}0& {\boldsymbol{\upgamma}}^{\mathrm{T}}\\ {}\boldsymbol{\upgamma} & {K}_i\left({\mathbf{x}}_i,\mathbf{x}\right)+I\end{array}\right] $$ is nonsingular, Eq. (20) can be converted to Eq. (21) by small transformation as follows:

21 $$ \left[{b}_i\kern0.5em {\mathbf{a}}_i\right]=\left[\begin{array}{cc}0& {\mathbf{y}}_i\end{array}\right]{\left[\begin{array}{cc}0& {\boldsymbol{\upgamma}}^{\mathrm{T}}\\ {}\boldsymbol{\upgamma} & {K}_i\left({\mathbf{x}}_i,\mathbf{x}\right)+I\end{array}\right]}^{-1} $$

Then, MLSSVM approximation is expressed as:

22 $$ {f}_i\left(\mathbf{x}\right)={\mathbf{a}}_iK\left({\mathbf{x}}_i,{\mathbf{x}}_j\right)+{b}_i $$

The MLSSVM identification of the extended inverse model for soft-sensing depends heavily on the selection of kernel function parameter **σ** and regularization parameter **γ**. If **σ** is too small, the training effect will be undermined by the localized kernel; Otherwise, there will be a high risk of undertraining; If **γ** is too small, the training error will increase and the learning machine will have stronger generalization ability; Otherwise, the training error will decrease and the learning machine will have a weaker generalization ability. Traditionally, these parameters are selected empirically through trial-and-error. The selection process is inaccurate and time-consuming. To ensure the prediction precision of our extended inverse model, this paper adopts the ABC algorithm to optimize and fine-tune the parameter combination (**σ**, **γ**).

### ABC optimization algorithm

In recent years, the research on intelligent optimization algorithm and its application in model parameter optimization is very active and has achieved encouraging results [15, 17, 18]. Inspired by the foraging behavior of bees, the ABC is an intelligent optimization algorithm that has been successfully applied in optimization of model parameters [19, 20]. This algorithm does not care about the specific information of the problem, but the merits and demerits of the problem. By the ABC, three types of bees are set up to perform local optimization, and the optimal food source is updated iteratively to obtain the global optimal solution. Therefore, the ABC converges fast and stays immune to the local optimum trap, providing an effective way to solve multi-dimensional engineering problems. Many numerical examples have shown that the ABC has better optimization and convergence performance than differential evolution (DE), genetic algorithm (GA) and particle swarm optimization (PSO) [21, 22]. That is why this algorithm is adopted here to optimize two key parameters. Based on this, the paper selects the ABC algorithm to optimize the performance parameters of MLSSVM.

In the ABC, the colony consists of three groups of bees: leading bees, following bees and scout bees. The leading bees whose food source has been abandoned becomes a scout. The leading bees search for high-quality food sources, the following bees watch the dances of leading bees and choose one source depending on the dances, and the scout bees search for new food sources randomly around the chosen source. The total number of leading bees and following bees equals the number of food sources. Let *S*_*N*_. *x*_*ij*_(*i* = 1, 2, ⋯, *SN*, *j* = 1, 2, ⋯, *D*) be the locations of food sources, with *D* be the number of optimization parameters. After initialization, the leading bees start to search for food sources iteratively. In each iteration, an leading bee remembers the new food source, if it has a higher nectar amount than the old one. The following bees will choose a food source, go to that source, choose a neighbor, and evaluate its nectar amount. Then, abandoned food sources are determined and are replaced with the new food sources discovered scout bees. Finally, the best food source found so far is registered.

The leading bees search for new solutions based on their current location, which can be described as follows:

23 $$ {v}_{ij}={x}_{ij}+{\phi}_{ij}\left({x}_{ij}-{x}_{kj}\right) $$

where: *k* ∈ {1, 2, ⋯, *S*_*N*_} and *j* ∈ {1, 2, ⋯, *D*} are randomly selected, and *k* ≠ *i*. *ϕ*_*ij*_ is a random number between [−1, 1].

The conversion probability of each individual is calculated as follows:

24 $$ {P}_i=\frac{f\left({x}_i\right)}{\sum \limits_{n=1}^{S_N}f\left({x}_n\right)} $$

where: *f*(**x**_*i*_) is the fitness value of each individual and *S*_*N*_ is the number of food sources.

If the solution *x*_*i*_ update fails, it means that the solution can not be optimized and needs to be replaced by a new solution generated by running the following formula:

25 $$ {\mathbf{x}}_{ij}={\mathbf{x}}_{\min, j}+\mathit{\operatorname{rand}}\left(0,1\right)\left({\mathbf{x}}_{\max, j}-{\mathbf{x}}_{\min, j}\right) $$

**Fig. 3:**
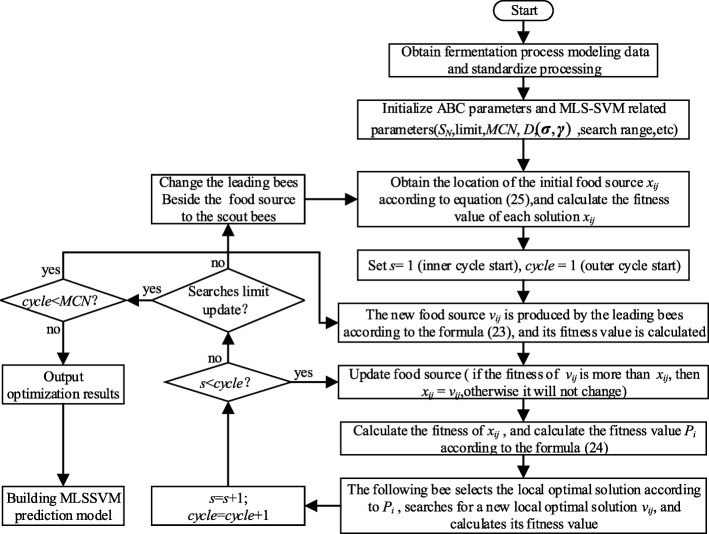
According to the deviation between the off-line, assay analysis value in the actual fermentation process and the output of the inversion soft-sensing model, the ABC algorithm is used to optimize and adjust the performance parameters of the MLS-SVM, so that the initial inverse expanded model can be corrected on-line

When using the ABC algorithm to optimize the MLSSVM parameters, it is necessary to set the relevant parameters and fitness functions, including the initialization of the control parameters in the ABC algorithm. The detailed flow chart of ABC-MLSSVM is shown in Fig. 3, the specific parameters are set as follows:

1. Initialize various parameters in the ABC algorithm, the number of food source *S*_*N*_ is 20, the maximum number of searches limit is 50, and the number of termination cycles *MCN* is 100.
2. Performance parameters (**γ**, **σ**) of MLSSVM represents the location of food source, *D* is set to 6, and the search range of LSSVM parameters is set to [0.01, 1000].
3. Set the usage function in the ABC algorithm. The purpose of optimizing MLSSVM is to reduce the prediction error, so the fitness function is applied as $F\left({x}_i\right)=\frac{1}{MS{E}_i}$, where *MSE*_*i*_ represents the root mean square error of the MLSSVM of *i*-th solution.

ABC optimization algorithm does not care about the specific information of the problem, but the merits and demerits of the problem. It can quickly converge and keep immune to the local optimal trap, which provides an effective way to solve multi-dimensional engineering problems. Based on this, this paper uses ABC algorithm to optimize the performance parameters (**σ**, **γ**) of MLSSVM, in order to get a more accurate inverse model.

### Inverse model identification based on ABC-MLSSVM

**Fig. 4:**
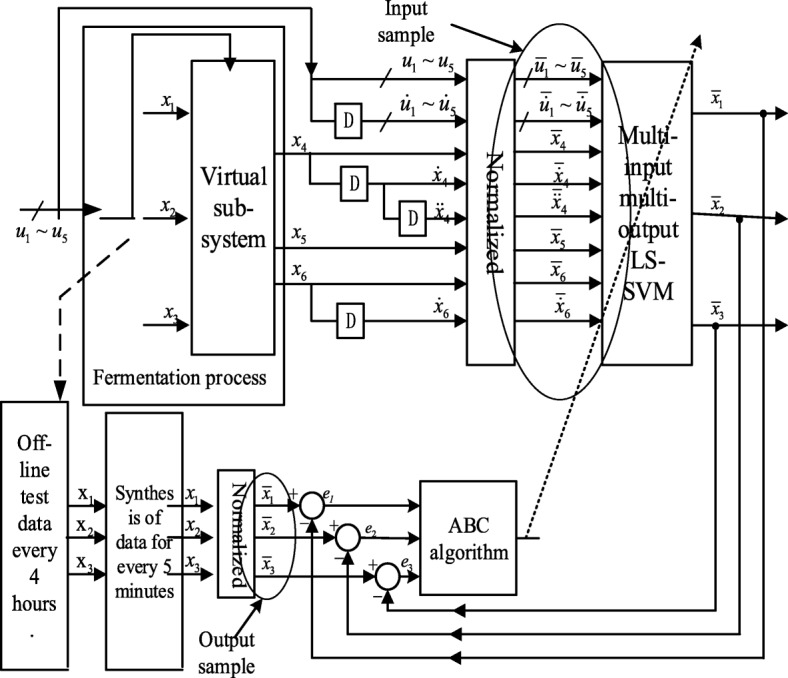
The initial value is preset to initialize the ABC algorithm, and the dimension of variables and boundary conditions are set. The fitness function is applied as *F*(*x*_*i*_) = 1/*MSE*_*i*_, where *MSE*_*i*_ represents the root mean square error of the MLS-SVM of *i* th solution, *MSE*_*i*_ of the initial MLS-SVM training sample is calculated as the initial value of the fitness function, and the minimum value of the fitness function in the global range is the optimal MLS-SVM soft-sensing model, in this case, the corresponding (**γ**, **σ**) is the optimal solution in the set search range

The order of each input and its derivative of ABC-MLSSVM inverse soft-sensing model is determined according to Eq. (14), and *φ*_4_, *φ*_5_, *φ*_6_ in Eq. (14) are obtained by using MLSSVM off-line identification and ABC algorithm on-line optimization. Then the inverse soft-sensing model developed in this way can realize the soft-sensing of *x*_1_, *x*_2_, *x*_3_, and the kernel function of MLSSVM is Gauss radial basis function. The identification process of inverse soft-sensing model is as follows:

1. Fermentation data acquisition. On the premise of meeting the sampling theorem, the input variable **u** is collected with appropriate excitation signal during the working area of the marine low-temperature alkaline protease MP fermentation process, direct measurable parameter {*x*_4_, *x*_5_, *x*_6_}, and process parameter {*W*_*t*_, *P*_*t*_, *S*_*a*_, *F*_*a*_} to obtain the original data sample set {*u*_1_, *u*_2_, *u*_3_, *u*_4_, *u*_5_, *x*_4_, *x*_5_, *x*_6_, *W*_*t*_, *P*_*t*_, *S*_*a*_, *F*_*a*_}. Non-direct measurable variable {*x*_1_, *x*_2_, *x*_3_} can be obtained by off-line, assay analysis in the laboratory.
2. Data preprocessing. Through certain technical processing (such as digital filtering, improving measurement redundancy, etc.), the bad data caused by working conditions, manual operation or environmental impact can be deleted, and the reliability of sample data can be improved. At the same time, in order to accurately calculate the required derivatives, according to the structure of the extended inverse model determined by Eq. (14), the five-point derivation method is adopted to obtain the every derivative $\left\{{\dot{x}}_4,{\ddot{x}}_4,{\dot{x}}_5,\dot{\mathbf{u}}\right\}$ of {*x*_4_, *x*_5_, **u**}, and the interpolation method is used to process the {*x*_1_, *x*_2_, *x*_3_} (keep it synchronized with measurable data in real time to ensure consistency of data), and finally the data sample sets {*x*_1_, *x*_2_, *x*_3_} and $\left\{{x}_4,{x}_5,{x}_6,{\dot{x}}_4,{\ddot{x}}_4,{\dot{x}}_5,\mathbf{u},\dot{\mathbf{u}},{W}_t,{P}_t,{S}_a,{F}_a\right\}$ are obtained, the former is used as the output of the inverse soft-sensing model, that is, the key biological parameter, and the latter is the input of the inverse soft-sensing model.
3. Off-line training and on-line correction. According to the input and output sample data, the MLSSVM is trained off-line and the corresponding initial parameters are determined by cross-validation, and the initial inverse expanded model is established. Then, according to the deviation between the off-line, assay analysis value in the actual fermentation process and the output of the inversion soft-sensing model, the ABC algorithm is used to optimize and adjust the performance parameters of the MLSSVM, so that the initial inverse expanded model can be corrected on-line. Figure 4 shows the on-line correction figure of the inverse soft-sensing model of fermentation process.

## Results

**Fig. 5:**
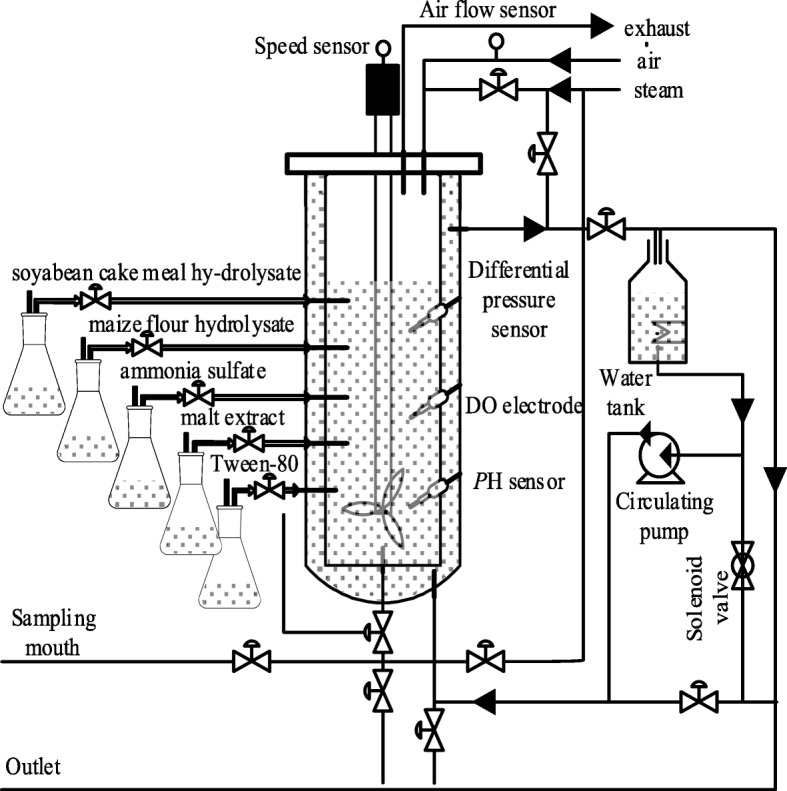
The fermentation tank is added into the culture medium (soyabean cake meal hy-drolysate, maize flour hydrolysate, ammonia sulfate, malt extract, Tween-80) and sterilized by steam at high temperature, after cooling, the fermentation strain is connected to the fermentation strain in a certain proportion, and the appropriate amount of the enzyme producing strain is carried out at the right time

Take the low-temperature alkaline protease MP fermentation process as the object for experimental verification. The flow chart of marine low-temperature alkaline protease MP fermentation process is shown in Fig. 5. In order to make the experiment closer to the production process, the experiment scheme is designed as follows:

1. The high-yield low-temperature alkaline protease strain YS-80 isolated from Huang Hai water samples of China is selected as the strain (with the characteristics of short fermentation period, high protease yield, good enzyme stability, safety and reliability, non-toxicity and so on). It is fermented in the fermenter of 1m^3^ and is fermented according to the technological requirements of the marine alkaline protease MP fermentation. After the fermentation tank is added into the culture medium and sterilized by steam at high temperature, after cooling, the fermentation strain is connected to the fermentation strain in a certain proportion, and the appropriate amount of the enzyme producing strain is carried out at the right time.
2. Set fermentation period *T* as 90 h and sampling period *t* as 5 min of each batch, the fermentation temperature is controlled at about 28 °C, the pH value is about 9.5, the tank pressure is controlled at 0.04 Mpa, the stirring speed is controlled at 250r/min, the dissolved oxygen is controlled between 45~75%, and ventilation volume is 1000 L/h. Non-direct measurable variable {*x*_1_, *x*_2_, *x*_3_} is obtained by off-line analysis and test after regular sampling (the appropriate fermentation broth is taken every 4 h through sampling mouth) in the laboratory. Among them, *X* is obtained based on the cell dry weight method, a certain amount of fermentation broth is centrifuged at 3000r/min for 5min in a centrifuge tube. Then, the supernatant is discarded, washing twice with distilled water, and drying it at 105 °C to a constant weight, weighing it. *S* is measured using a SBA − 40A glucose analyzer and *P* is determined by an automatic scanning spectrophotometer.
3. Only 10 batches of sample data are considered to test the identification ability of ABC-MLSSVM inverse soft-sensing model to small samples in the experiment. In order to enhance the difference among different batches, the initial conditions of each batch fermentation and the feeding strategy of each nutrient solution are set to be different. And the first six batches of fermentation data are used as training samples to off-line train the inverse expanded model of fermentation process. The seventh batch and the eighth batch of fermentation data are used to on-line correction the initial extended inverse model, and the ninth batch and the 10th batch fermentation data are used to verify the effectiveness and prediction accuracy of the inverse expanded model.

In order to test the performance of the ABC-MLSSVM inverse soft-sensing modeling method, it is compared with the traditional LSSVM soft-sensing modeling method, and the relative errors of the prediction results of the two methods are calculated. The initial performance parameters of MLSSVM are taken as: **σ**^2^ = [1.0, 1.0, 1.0], **γ** = [10, 10, 10], and the performance parameters of MLSSVM after on-line optimization by ABC algorithm are **γ** = [10.1, 6.3, 8.2], **σ**^2^ = [0.532, 1, 613, 0.479].

**Fig. 6:**
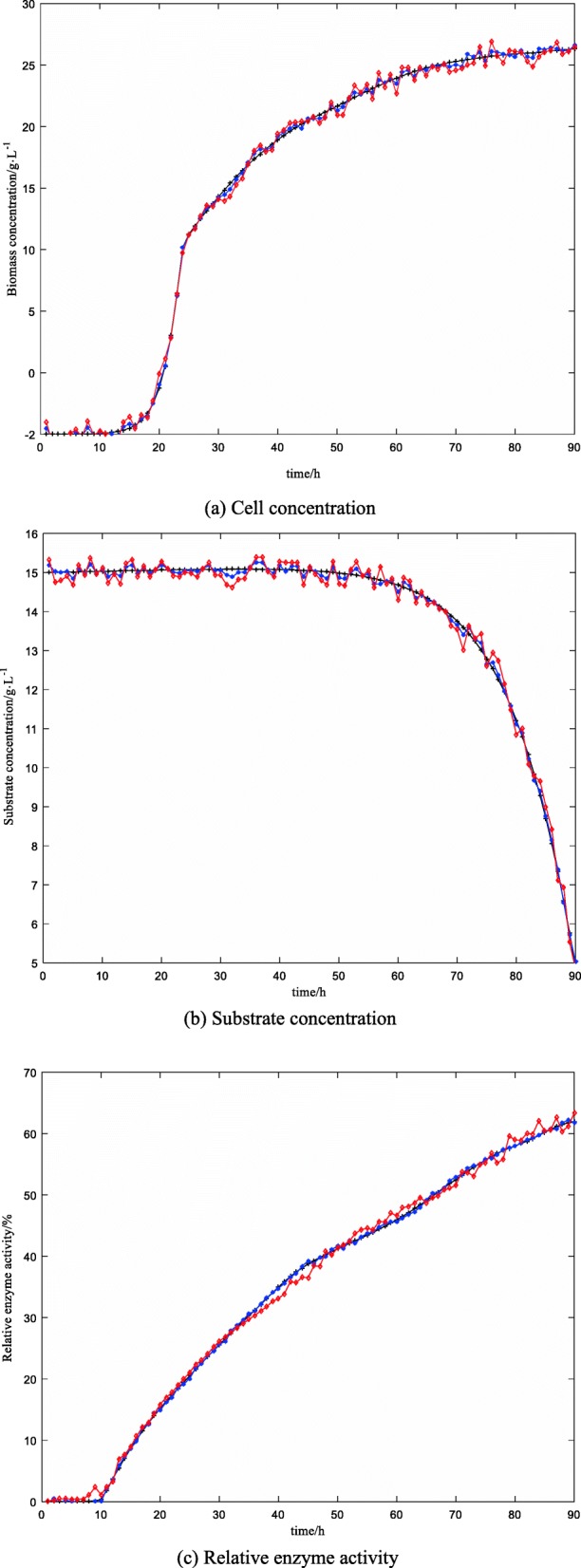
The actual data curve is described by a black solid line with +, the predictive value curve of ABC-MLSSVM inversion soft-sensing modeling is described by a red solid line with *, the predictive value curve of MLSSVM soft-sensing modeling is described by a blue solid line with ◊. (**a**) shows the comparison between the the predictive value curve of biomass concentration and the actual data curve. (**b**) shows the comparison between the the predictive value curve of substrate concentration and the actual data curve. (**c**) shows the comparison between the the predictive value curve of relative enzyme activity and the actual data curve

**Fig. 7:**
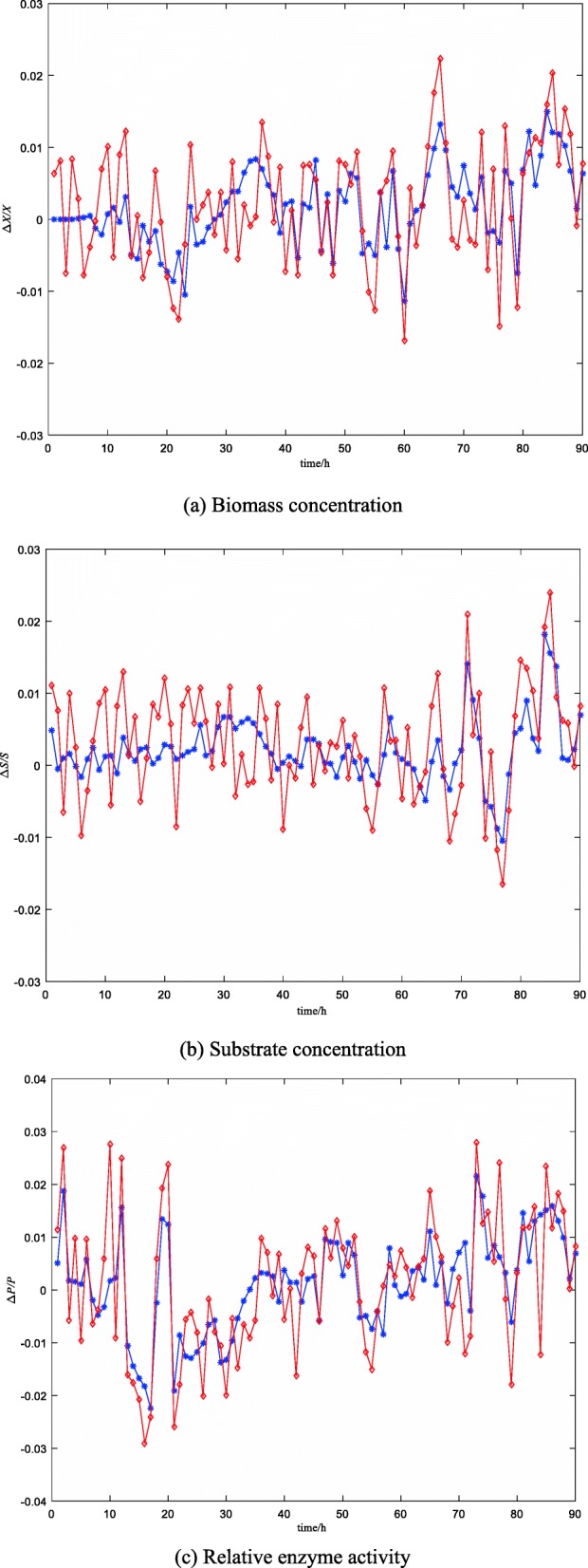
The relative error curve of ABC-MLSSVM inversion soft-sensing modeling is described by a red solid line with *, the relative error curve of MLSSVM soft-sensing modeling is described by a blue solid line with ◊. (**a**) is the relative error curve of biomass concentration. (**b**) is the relative error curve of substrate concentration. (**c**) is the relative error curve of relative enzyme activity

Figure 6 is a comparison of soft-sensing results of key biological parameters of the ninth batch fermentation (protease content is characterized by relative enzyme activity in Fig. 6). Figure 7 is a relative error curve. Table 1 lists the average relative error MRE of the soft-sensing results of the two methods (protease content is characterized by relative enzyme activity in Fig. 7).

**Table 1:** MRE comparison by two models

Fermentation batch	ABC-MLSSVM Inversion			MLSSVM		
	*X*/g·L^− 1^	*S*/g·L-1	*E*/g·L-1	*X* /g·L-1	*S*/g·L-1	E /g·L-1
The 9th batch	1.38%	1.63%	1.97%	4.62%	3.36%	5.76%
The 10th batch	1.43%	1.72%	2.06%	5.11%	2.98%	6.32%

## Discussion

As can be seen from Figs. 6, 7 and Table 1, compared with the traditional LS-SVM soft-sensing method, the on-line estimation results of the ABC-MLSSVM inverse soft-sensing method are closer to the off-line assay values, especially in the prediction of cell concentration. It is fully proved that the reversibility assumption of the “virtual sensor” is reasonable. During the logarithmic growth period and stable growth period (20 h–60 h) of marine low-temperature alkaline protease MP fermentation, the average RMSE (root-mean-square error) of cell concentration, substrate concentration and relative enzyme activity are 0.146, 0.127 and 0.185 respectively when the MLS-SVM method is used. While when the ABC-MLSSVM inversion method is adopted, the soft-sensing RMSE of the there results are 0.0645, 0.0538 and 0.0712. This indicates that the ABC-MLSSVM inverse system method is effective and credible, and can greatly improve the soft-sensing precision of key biological parameters in the low-temperature alkaline protease MP fermentation process, which satisfactorily meets the expected accuracy requirements.

## Conclusion

In order to solve the problem that the key biological parameters of marine low-temperature alkaline protease MP cannot be measured directly on-line during fed-batch fermentation, a soft-sensing modeling method for marine low-temperature alkaline protease MP fermentation process based on ABC-MLSSVM inversion is proposed by combining the inverse system method with least square support vector machine. This paper firstly establishes a “gray-box” model for the marine low-temperature alkaline protease MP fermentation process based on the material balance. Then, the reversibility of the nonlinear model was analyzed based on the inverse method, and the extended inverse model was constructed, coupling MLSSVM system identification with ABC optimization. Finally, the extended inverse model was connected in series with the original fermentation system, forming a composite pseudo-linear system. The composite system supports the online prediction of key biological parameters in fermentation process. The simulation results show the rationality of the system dynamic model and the validity of ABC-MLSSVM inverse soft-sensing method for predicting the key biological parameters of marine low-temperature alkaline protease MP fermentation process.

The proposed model offers a feasible theoretical method to solve the soft-sensing of key biological parameters of the marine alkaline protease MP fermentation process. The model achieves ideal identification effect based on a few input/output data, eliminating the need for an exact kinetics model of the fermentation process. The soft-sensing of key parameters can be achieved by connecting the inverse system with the original system into a composite system. With clear physical meanings, The ABC-MLSSVM inversion soft-sensing method effectively overcomes the bottleneck of traditional inverse system method: The difficulty in implementing an accurate model, and enables the soft-sensing of general nonlinear reversible systems. The proposed model enjoys a wide scope of applications, laying the basis for nonlinear soft-sensing modelling of MIMO systems.

## Data Availability

The datasets used and/or analysed during the current study are available from the corresponding author on reasonable request.

## Abbreviations

ABC: Artificial bee colony
CL: Dissolved oxygen concentration
E: Relative enzyme activity
LS-SVM: Least square support vector machine
MLSSVM: Multiple least squares support vector machine
S: Substrate concentration
V: Fermentation broth volume
X: Cell concentration protease content
